# Hypotheses, rationale, design, and methods for evaluation of ischemic preconditioning assessed by sequential exercise tests in diabetic and non-diabetic patients with stable coronary artery disease – a prospective study

**DOI:** 10.1186/1471-2261-13-117

**Published:** 2013-12-13

**Authors:** Paulo Cury Rezende, Rosa Maria Rahmi Garcia, Augusto Hiroshi Uchida, Leandro Menezes Alves Costa, Thiago Luis Scudeler, Rodrigo Morel Vieira Melo, Fernando Teiichi Costa Oikawa, Cibele Larrosa Garzillo, Eduardo Gomes Lima, Carlos Alexandre Wainrober Segre, Desiderio Favarato, Priscyla Girardi, Myrthes Takiuti, Celia Cassaro Strunz, Whady Hueb, José Antonio Franchini Ramires, Roberto Kalil Filho

**Affiliations:** 1 Department of Atherosclerosis, Heart Institute of the University of Sao Paulo, Sao Paulo, Brazil

**Keywords:** Ischemic preconditioning, Exercise test, Coronary heart disease, Angina, Myocardial ischemia

## Abstract

**Background:**

Ischemic preconditioning is a powerful mechanism of myocardial protection and in humans it can be evaluated by sequential exercise tests. Coronary Artery Disease in the presence of diabetes mellitus may be associated with worse outcomes. In addition, some studies have shown that diabetes interferes negatively with the development of ischemic preconditioning. However, it is still unknown whether diabetes may influence the expression of ischemic preconditioning in patients with stable multivessel coronary artery disease.

**Methods/Design:**

This study will include 140 diabetic and non-diabetic patients with chronic, stable coronary artery disease and preserved left ventricular systolic function. The patients will be submitted to two sequential exercise tests with 30-minutes interval between them. Ischemic parameters will be compared between diabetic and non-diabetic patients. Ischemic preconditioning will be considered present when time to 1.0 mm ST-segment deviation is greater in the second of two sequential exercise tests. Exercise tests will be analyzed by two independent cardiologists.

**Discussion:**

Ischemic preconditioning was first demonstrated by Murry et al. in dog’s hearts. Its work was reproduced by other authors, clearly demonstrating that brief periods of myocardial ischemia followed by reperfusion triggers cardioprotective mechanisms against subsequent and severe ischemia. On the other hand, the demonstration of ischemic preconditioning in humans requires the presence of clinical symptoms or physiological changes difficult to be measured. One methodology largely accepted are the sequential exercise tests, in which, the improvement in the time to 1.0 mm ST depression in the second of two sequential tests is considered manifestation of ischemic preconditioning.

Diabetes is an important and independent determinant of clinical prognosis. It's a major risk factor for coronary artery disease. Furthermore, the association of diabetes with stable coronary artery disease imposes worse prognosis, irrespective of treatment strategy. It’s still not clearly known the mechanisms responsible by these worse outcomes. Impairment in the mechanisms of ischemic preconditioning may be one major cause of this worse prognosis, but, in the clinical setting, this is not known.

The present study aims to evaluate how diabetes mellitus interferes with ischemic preconditioning in patients with stable, multivessel coronary artery disease and preserved systolic ventricular function.

## Background

Ischemic preconditioning (IP) is a physiologic phenomenon of tissue protection observed in many organs, including the heart, in which brief periods of repetitive ischemia can protect cells from the corresponding area during a subsequent ischemic insult. IP was first demonstrated by Murry et al. in 1986 in canine hearts [1]. Through the interruption in circumflex artery flow for 5 minutes, followed by 5 minutes of reperfusion, the authors promoted 4 cycles of ischemia/reperfusion before a prolonged ischemic insult of 40 minutes. When compared to the control group, in which these brief ischemic stimuli were not performed, the “preconditioned” group presented a 75% reduction in the infarct area. This first work was then reproduced by other authors, with similar results. This phenomenon of myocardial protection is considered the most powerful known mechanism of cardioprotection discovered so far.

The IP-related cardioprotection in the human myocardium can be observed during percutaneous coronary angioplasty after coronary occlusion with balloons [2], during coronary artery bypass surgery with intermittent clamping of the aorta [3] and in clinical studies [4,5].

Although ischemic preconditioning can be easily demonstrated in experimental studies, in humans this is more complex due to the fact that one needs to promote myocardial ischemia in order to trigger the mechanisms of cardioprotection. One methodology widely accepted by literature is the sequential exercise tests (SET) [6-10]. During these tests, ischemic parameters like the time to develop 1.0 mm ST depression in an electrocardiogram print-out, the time to develop angina, the morphology of ST depression, the total exercise time and the double-product at the time of 1.0 mm ST-depression are registered in these two sequential tests and compared [9]. Patients are considered to present ischemic preconditioning if they improve ischemic parameters in the second of two tests [9,11]. The most widely accepted parameter to consider the presence of IP is the improvement in the time to 1.0 mm ST depression [11,12].

In patients with chronic coronary artery disease (CAD), diabetes is strongly associated with worse outcomes [13]. And this is irrespective of the treatment applied [14]. Although these worse prognosis is not totally understood, some studies infer that diabetes may negatively interfere with the development of IP [15,16]. On the other hand, experimental studies have demonstrated equivocal results [17]. Thus, it`s still unknown whether diabetes can influence the expression of IP in patients with stable multivessel CAD.

## Methods/Design

This study is an institutional project conducted at the Heart Institute (InCor), Clinics Hospital, University of Sao Paulo, and is designed to prospectively investigate the expression of ischemic preconditioning in 140 diabetic and non-diabetic patients with stable, multivessel coronary artery disease, and preserved ventricular function. Of note, IP will be assessed by sequential exercise tests and compared between diabetic and non-diabetic patients.

**Figure 1:**
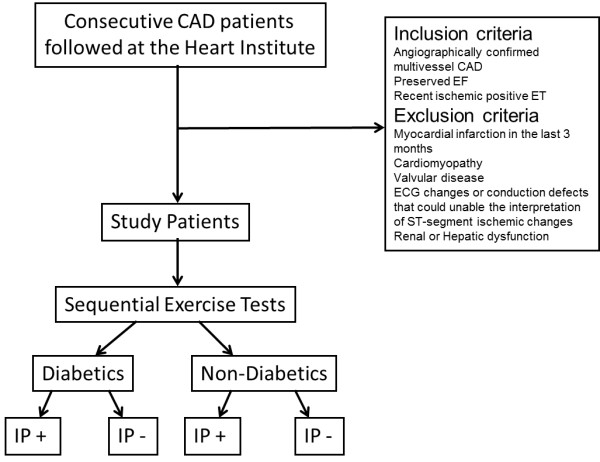
Flowchart of Study Design.

This study is designed as a prospective cohort study, in which patients with angiographically confirmed coronary artery disease, preserved systolic ventricular function and a recent ischemic treadmill exercise test will be included. Before inclusion, all patients will undergo an outpatient clinical evaluation. After inclusion, they will be submitted to two sequential exercise tests, with a 30-minutes interval between them. Ischemic parameters will be registered and compared. IP will be assessed by an improvement is ischemic parameters in the second of two exercise tests, and its manifestation and intensity will be compared between patients with diabetes and patients without diabetes mellitus (Figure 1).

### Patients

Consecutive patients followed in outpatient visits in our Institution will be screened for inclusion and exclusion criteria. Patients with angiographically confirmed multivessel coronary artery disease (internal diameter reduction ≥70% of at least two major coronary branches), stable symptoms (Canadian class angina I-II), preserved left systolic ventricular function confirmed by transthoracic echocardiography (left ventricular ejection fraction ≥50%), and a recent positive exercise stress test for myocardial ischemia (horizontal or downsloping ST-segment depression ≥1.0 mm) will be enrolled. Patients will not be included if they present evidence of left ventricular hypertrophy, myocardial infarction in the last 3 months, cardiomyopathy, valvular disease, electrocardiogram changes, or conduction defects that could unable the interpretation of ST-segment ischemic changes, or renal or hepatic dysfunction.

After enrollment, patients will be discouraged from activities likely to induce angina or that require the symptomatic relief of short-acting nitrates. They will also be advised to not use any anti-diabetic or cardiovascular medications for 5 days before the exercise tests protocol. Only nitrates will be permitted in order to control ischemic symptoms, but they will be withdrawn 12 h before treadmill exercise tests. They will also be instructed not to take caffeine for 24 h before their test. They will have a 24-hour telephone number to contact study physicians in case of worsening symptoms or any doubts on medications.

### Treadmill exercise tests

All patients will be submitted to computer-assisted treadmill exercise tests, symptom limited, using the Bruce protocol, with a recovery phase of 5 minutes. The time interval between the consecutive tests will be 30 minutes. A 12-lead electrocardiogram, heart rate and arterial blood pressure will be obtained with the patient in standing position at baseline. A 12-lead ECG will also be obtained at each 1.0-min interval during exercise, at peak exercise, each minute up to 5 min after the exercise phase, at the onset of 1.0 mm ST-segment depression, at major ST-segment deviation, at the onset of angina pectoris, and when clinically relevant. The level of the ST-segment deviation will be based on visual analyses of the 0.08 s after the J point by two independent cardiologists in a blind fashion. In case of disagreement, a third cardiologist will be consulted and the matter will be resolved by consensus. Only horizontal or downsloping ST-segment depressions will be considered for the time to the onset of 1.0 mm ST-segment depression evaluation (T-1.0 mm). Criteria for interrupting the exercise test will be limiting chest pain, physical exhaustion, ST-segment depression ≥3.0 mm, ST-segment elevation ≥2.0 mm, maximum age-related heart rate, severe arterial hypertension, severe arterial hypotension, and complex or sustained arrhythmias. The following parameters will be measured in all patients: resting heart rate and arterial blood pressure, heart rate and arterial blood pressure at peak exercise, T-1.0 mm in seconds, rate-pressure product (RPP) at the onset of ST-segment depression and exercise duration in seconds.

### IP analysis

The improvement in ischemic parameters in the second test compared to the first one will indicate the presence of IP. The parameters that will be used to evaluate IP will be: T-1.0 mm and RPP. An improvement of at least 30 s in T-1.0 mm between the two sequential tests will be used to consider IP present, as previously described [11]. In the case of an improvement less than 30 s, an improvement in RPP will also be used to indicate the presence of IP.

### Statistical analysis

All data will be expressed as means ± standard deviation or median ± interquartile range, as appropriate. For categorical variables, the Fisher`s exact test will be used. Continuous variables not distributed normally, as evaluated by the Kolmogorov-Smirnov test, will be compared by the Mann–Whitney test. Continuous variables with a normal distribution will be compared by the Student’s t test. All tests will be 2-tailed, and statistical significance will be assumed when P < 0.05. Statistical analyses will be performed using the software SPSS, version 17.0.

### Sample size

The aim of the present study will be to detect differences in the occurrence and magnitude of IP assessed by sequential treadmill exercise tests, using T-1.0 mm in diabetic and non-diabetic patients as the main parameter evaluated. Therefore, the sample size calculation is based on previous studies’ differences in IP between groups of diabetic and non-diabetic patients. Based on these studies, the observed improvement in time to 1.0 mm ST-deviation in diabetic patients was 87 s ± 44 s [18] and the improvement in non-diabetic patients was 123 ± 65 s [19]. Thus, accounting for a loss of 30% of patients who may not express IP (based on the experience of our research group), 70 patients with diabetes and 70 patients without diabetes need to be studied to be able to test the null hypothesis that the population means are equal (power = 0.9 and alpha = 0.05).

## Discussion

There is no study in humans so far which have assessed the differences in IP between patients with and without diabetes and chronic coronary artery disease.

Diabetes is a known clinical condition associated with the development of ischemic heart disease [20], and in patients with established cardiovascular diseases, diabetes confers a worse prognosis [13,14]. Besides the fact that diabetes interferes in many stages in the development and progression of coronary artery disease, it`s postulated that diabetic patients seem to be more susceptible to ischemic injury [21,22]. Although some studies with humans during percutaneous coronary intervention [15] or during the clinical context of an acute myocardial infarction [16] show that diabetes interferes negatively with IP, experimental studies have demonstrated conflicting results [17]. However, the comparison of these results is controversial by many reasons, and mostly by the duration, severity and the type of diabetes and by different study protocols. Besides, end points also differ between studies, as well as the protocol of ischemia, the intensity of ischemia (zero flow versus low-flow ischemia) and the presence of the reperfusion phase. Furthermore, differences between species in relation to coronary collaterals and in myocardial perfusion may contribute to the variability of the results.

Moreover, these differences between clinical and experimental studies may be due to the contribution of other diseases in diabetic patients, such as hypertension, dislypidemia, microvascular dysfunction, neuropathy, nephropathy, as well as the use of antidiabetic drugs which may block IP by preventing the opening of K(ATP) channels in the myocardium [23,24]. The interaction among all these conditions may determine the overall response of the myocardium to ischemia in patients with diabetes mellitus.

The complex physiopathology of diabetes associated with interactions with its associated comorbidities, among them the aterosclerotic macrovascular disease, make this syndrome one of the greatest challenges of this century. The interaction of diabetes with cardioprotective mechanisms is of particular interest in clinical practice. Thus, the aim of the present study will be to analyze ischemic preconditioning in diabetic and non-diabetic patients with chronic, stable coronary artery disease by sequential treadmill exercise tests.

## Ethical considerations

This prospective study will be conducted in accordance with the principles of the Declaration of Helsinki and with laws and regulations of our country. The Ethics Committee of the Heart Institute of the University of São Paulo, Brazil approved the study protocol. The attending physician will obtain written informed consent from the study participants.

## Abbreviations

IP: Ischemic preconditioning; SET: Sequential exercise test; CAD: Coronary artery disease; ECG: Electrocardiogram; T-1.0 mm: Time to the onset of 1.0 mm ST-segment depression; RPP: Rate pressure product; ATP: Adenosine triphosphate.

## Competing interests

None of the authors of the present study has a financial or any other relation that would pose a conflict of interest.

## Author’ contributions

Each of the authors has made substantial contributions to this manuscript, either in the conception and design or in the drafting of the article and critical revision for important intellectual content. Specifically, study concept and design: PCR, RMRG, AHU, WH. Acquisition of data: PCR, RMRG, LMAC, TLS, RMVM, FTCO, CLG, EGL, CAWS, PG, MT, CCS. Analysis and interpretation of data: PCR, RMRG, AHU, LMAC, TLS, RMVM. Drafting of manuscript: PCR, AHU, WH. Critical revision of the manuscript for important intellectual content: PCR, AHU, WH, JAFR, RKF. Statistical expertise: DF, LCM. Study Supervision/Support and planned Ancillaries Studies: WH, LMAC, TLS, RMRG. All authors participated in drafting and revising the manuscript, and all authors have read and approved the final manuscript.

## Pre-publication history

The pre-publication history for this paper can be accessed here:

http://www.biomedcentral.com/1471-2261/13/117/prepub
